# Preservation of rat limbs by hyperbaric carbon monoxide and oxygen

**DOI:** 10.1038/s41598-018-25070-y

**Published:** 2018-04-26

**Authors:** Naoyuki Hatayama, Shuichi Hirai, Munekazu Naito, Hayato Terayama, Jun Araki, Hiroki Yokota, Masayuki Matsushita, Xiao-Kang Li, Masahiro Itoh

**Affiliations:** 1 Department of Anatomy, Aichi Medical University, Aichi, Japan; 2 Department of Anatomy, Tokyo Medical University, Tokyo, Japan; 3 Department of Anatomy, Division of Basic Medical Science, Tokai University School of Medicine, Kanagawa, Japan; 4 Department of Plastic and Reconstructive Surgery, Graduates School of Medicine, University of Tokyo, Tokyo, Japan; 5 Department of Molecular and Cellular Physiology, Graduate School of Medicine, University of the Ryukyus, Okinawa, Japan; 6 National Research Institute for Child Health and Development, Tokyo, Japan

## Abstract

Cold ischemia times ranging from <6 h to as long as 24 h are generally quoted as the limits for attempting the replantation of amputated extremities. In this study, we aimed to assess the effect of hyperbaric carbon monoxide (CO) and oxygen (O_2_) on rat limb preservation. Donor rat limbs were preserved in a chamber filled with hyperbaric CO and O_2_ for 3 days (CO + O_2_ 3 days) or 7 days (CO + O_2_ 7 days). Positive and negative control groups were created by using non-preserved limbs (NP) and limbs wrapped in saline-moistened gauze for 3 days (SMG 3 days), respectively. The survival rate of transplanted limbs at postoperative day 90 was 88% in the NP and 86% in the CO + O_2_ 3 days. The corresponding survival rate was 50% in the CO + O_2_ 7 days at postoperative day 90 but was 0% in the SMG 3 days at postoperative day 3. Muscle mass decreased in the CO + O_2_ 3 days and CO + O_2_ 7 days compared with the NP, but sciatic–tibial nerve conduction velocities did not differ. These results indicate that amputated extremities preservation with hyperbaric CO and O_2_ could extend the time limits of preservation, maintaining their viability for replantation.

## Introduction

Replantation is the surgical reattachment by microsurgical techniques of a body part—most commonly a finger, a hand, or an arm—that has been completely cut from the body. Recent technological advances and the use of microscopy have made the replantation of various anatomical parts possible, including thumbs, fingers, ears, scalps, facial parts, and genitalia^1–5^. As opportunities to replant have grown, it has become more important to preserve body parts for longer and in better conditions. To date, experiments on rats or rabbits have suggested that the cold ischemia time during limb preservation should not exceed 12–24 h^6–9^. Amputated extremities from humans are generally preserved by wrapping them in saline-moistened gauze (SMG), placing them in a plastic sealable bag, and putting the bag on ice^10^. Although successful digit replantation has been reported after 94 h, the recommended time limits for reliable success remain 24 h for digits and 12 h for major replants^11^. Given that accidents occur suddenly, and because time is needed to find a specialist to perform replantation, novel methods are needed to extend ischemia times when preserving amputated extremities for replantation^12^.

Carbon monoxide (CO) is a colorless, odorless, and tasteless gas that is toxic when it bonds with hemoglobin to form carboxyhemoglobin, preventing the delivery of oxygen (O_2_) to the tissues. However, CO also exerts vasoactive, antiproliferative, antioxidant, anti-inflammatory, and antiapoptotic effects and contributes substantially to cell protection^13,14^. Research has shown that these beneficial effects can somewhat prevent ischemia–reperfusion injury (IRI) following transplantation, with a stock solution of bubbling low-dose CO helping to preserve pig kidneys and rat livers^15,16^. It has also been shown that hyperbaric O_2_ reduces IRI that can worsen crush injuries, induce the compartment syndrome, and cause skin flap and reattachment failures by inhibiting the adherence of neutrophils that release proteases and produce free radicals^17^. However, hyperbaric O_2_ treatment also damages lung function, potentially causing cellular dysfunction. Clinically, hyperbaric O_2_ is used in very limited circumstances, such as after heart surgery, and extended periods of treatment are usually avoided^18^. Recently, we tried to preserve rat hearts in CO and O_2_ at high pressures and succeeded in preserving tissue for 24–48 h^19^. This success with organ preservation led us to consider whether the method might be effective for preserving amputated extremities.

We aimed to assess the efficacy of using CO and O_2_ under high pressures to preserve rat limbs. To do so, we transplanted preserved rat hind limbs to syngeneic recipient rats in which the corresponding limbs had been removed.

## Results

### Limb survival rates after preservation under different conditions

Donor rat limbs were preserved in a chamber filled with CO and O_2_ for 3 days (CO + O_2_ 3 days) or 7 days (CO + O_2_ 7 days) at 4 °C under high pressure. Positive and negative control groups were created by using non-preserved limbs (NP group) and limbs wrapped in SMG for 3 days at 4 °C (SMG 3 days), respectively.

**Figure 1:**
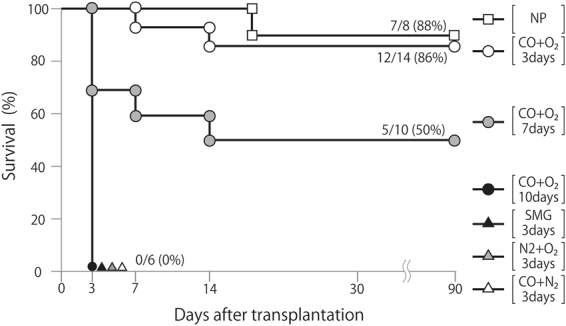
Survival rate after transplantation by preservation method. Limbs were preserved in chambers filled with CO + O_2_ for 3, 7, and 10 days, and CO + N_2_, or N_2_ + O_2_ for 3 days under high pressure. Negative control was achieved by wrapping limbs in saline-moistened gauze (SMG) for 3 days at 4 °C. For the positive control, non-preserved (NP) limbs were used from donor rats.

At postoperative day (POD) 7, the survival rate of transplanted limbs was 100% in the NP group, but graft failure occurred in one of those limbs because of self-harm and subsequent infection at POD 17 (*n* = 8; Fig. 1). The survival rate of transplanted limbs was 100% in the CO + O_2_ 3 days group at POD 7, but graft failure occurred in two of the limbs at POD 14 (*n* = 14; Fig. 1). At POD 3, the survival rate of transplanted limbs was 100% in the CO + O_2_ 7 days group, but there was graft failure in five limbs by POD 14 (*n* = 10; Fig. 1). All limbs in the groups treated with CO + O_2_ for 10 days, CO + N_2_ for 3 days, O_2_ + N_2_ for 3 days, and SMG for 3 days (*n* = 6 each) underwent necrosis by POD 3, precluding further evaluation. Graft survival was determined by macroscopic diagnosis and bleeding from the foot tip after (27-gage) needlestick injury. Graft failure was assumed in transplanted limbs when there was color to change black or bleeding was not present.

### Macroscopic and computed tomography images at POD 90

**Figure 2:**
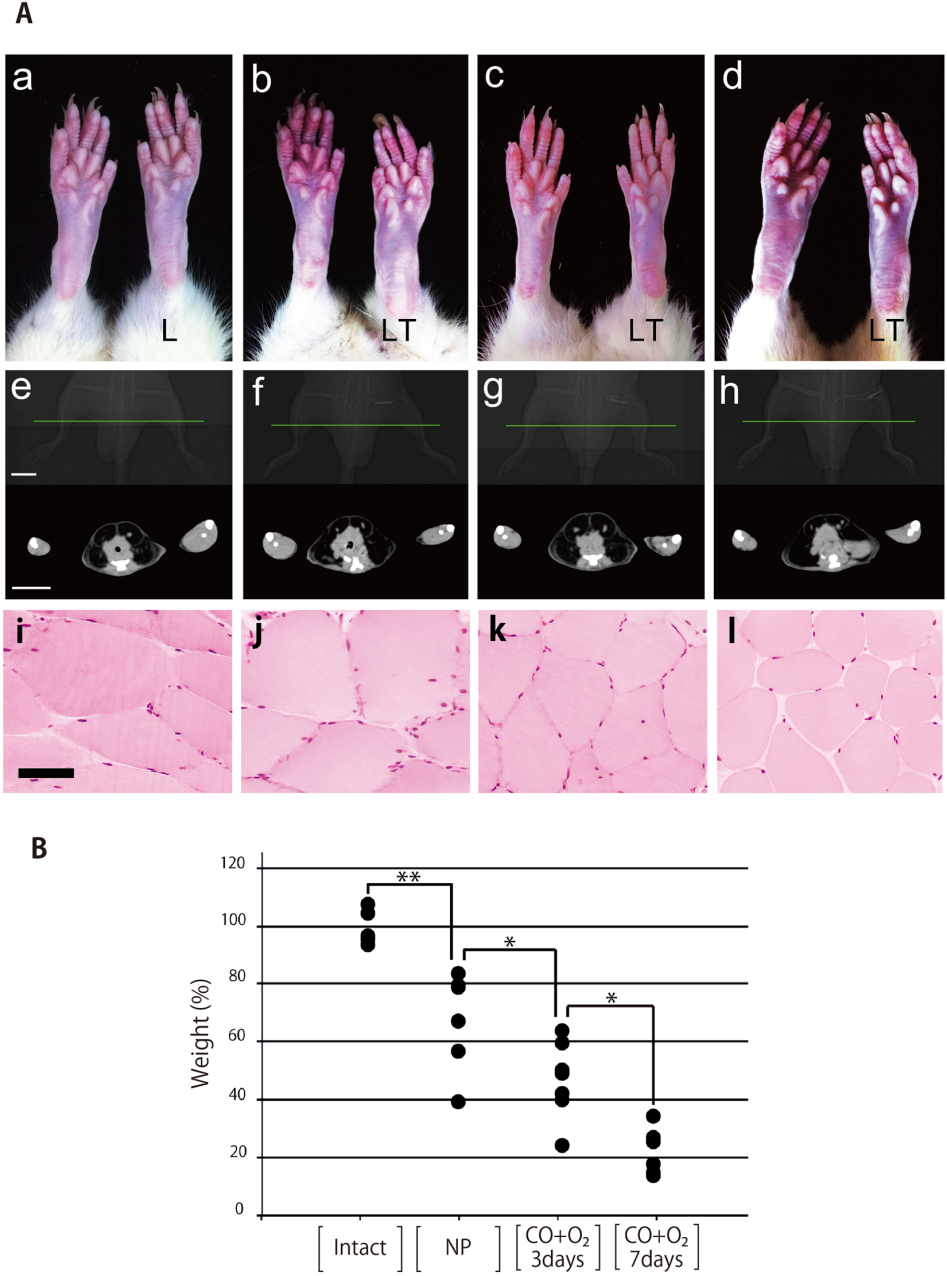
Muscle evaluation in transplanted limbs at postoperative day 90. **(A)** Microscopic views **(a**–**d)**, computed tomography images **(e**–**h)**, and histological images **(i**–**l)**. Intact** = a**, **e**, and **i**; non-preserved (NP) limbs = **b**, **f**, and **j**; 3 days of CO + O_2_ = **c**, **g**, and **k**; and 7 days of CO + O_2_ = **d**, **h**, and **l**. Circulation is normal in the transplanted left limbs in **a**, **b**, **c**, and **d**. Computed tomography images are shown above the green lines in the X-ray images from among each group. L, left hind limb. LT, left transplanted hind limb. White bar = 2 cm; Black bar = 100 μm. **(B)** Wet weights of triceps surae muscles. Data show the percentages of wet muscle weights in the transplanted left hind limbs compared with those in the non-transplanted right hind limbs per group. All data are expressed as means ± standard deviations, and analysis of variance (ANOVA) with the post-hoc Tukey–Kramer for statistical analysis. *P < 0.05 and **P < 0.01 by the Tukey–Kramer test.

Macroscopically, there were no observable differences in the skin of the non-transplanted right hind limb and the transplanted left hind limb in the NP, CO + O_2_ 3 days, and CO + O_2_ 7 days groups (Fig. 2A). Computed tomography showed slight atrophy of the triceps surae muscles of transplanted limbs in the NP, CO + O_2_ 3 days, and CO + O_2_ 7 days groups when compared with the intact limbs (Fig. 2A).

### Wet weights and histological images of the triceps surae muscles at POD 90

Wet muscle weight decreased significantly in descending order from the NP, CO + O_2_ 3 days, and CO + O_2_ 7 days groups, respectively, when compared with the Intact group (Fig. 2B). Atrophy of whole muscle fibers was observed in the CO + O_2_ 3 days and CO + O_2_ 7 days groups compared with the NP group but was more severe in the CO + O_2_ 7 days group than in the CO + O_2_ 3 days group (Fig. 2B).

### Sciatic–tibial nerve conduction velocities at POD 90

**Figure 3:**
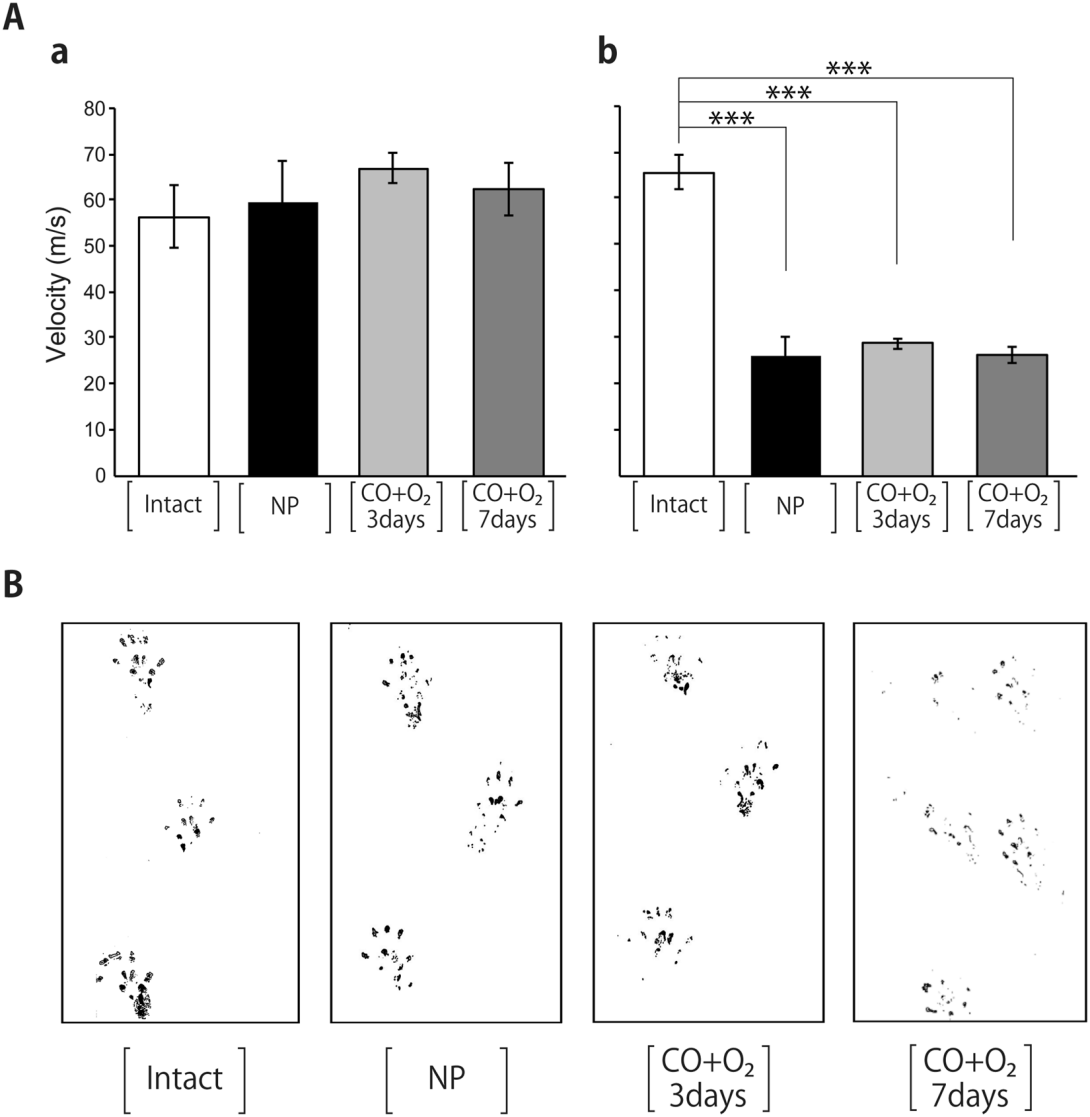
Neural activity in transplanted limbs at postoperative day 90. **(A)** Sciatic–tibial nerve conduction velocities. Motor nerve conduction velocities **(a)** and sensory nerve conduction velocities **(b)**. Data are shown per group and expressed as means ± standard deviations. The analysis of variance (ANOVA) with the Tukey–Kramer test post-hoc test was employed for statistical analysis. ***P < 0.001 by the Tukey–Kramer test. **(B)** Walking appearance. The black ink shows the representative walking track of each rat.

The sciatic–tibial motor nerve conduction velocity did not change in any of the groups (Fig. 3Aa) but did decrease compared with the Intact group in the NP, CO + O_2_ 3 days, and CO + O_2_ 7 days groups (Fig. 3Ab). However, no significant differences were found among the NP, CO + O_2_ 3 days, and CO + O_2_ 7 days groups (Fig. 3Ab).

### Walking appearance at POD 90

Walking appearance analysis showed that rats could walk using the transplanted limbs at POD 90 in the NP, CO + O_2_ 3 days, and CO + O_2_ 7 days groups (Fig. 3B). Foot stamps in the NP and the CO + O_2_ 3 days groups had almost the same appearances, but those in the CO + O_2_ 7 days group were obviously different.

### Preserving limbs in hyperbaric gases after initial wrapping in SMG for 6 h

**Figure 4:**
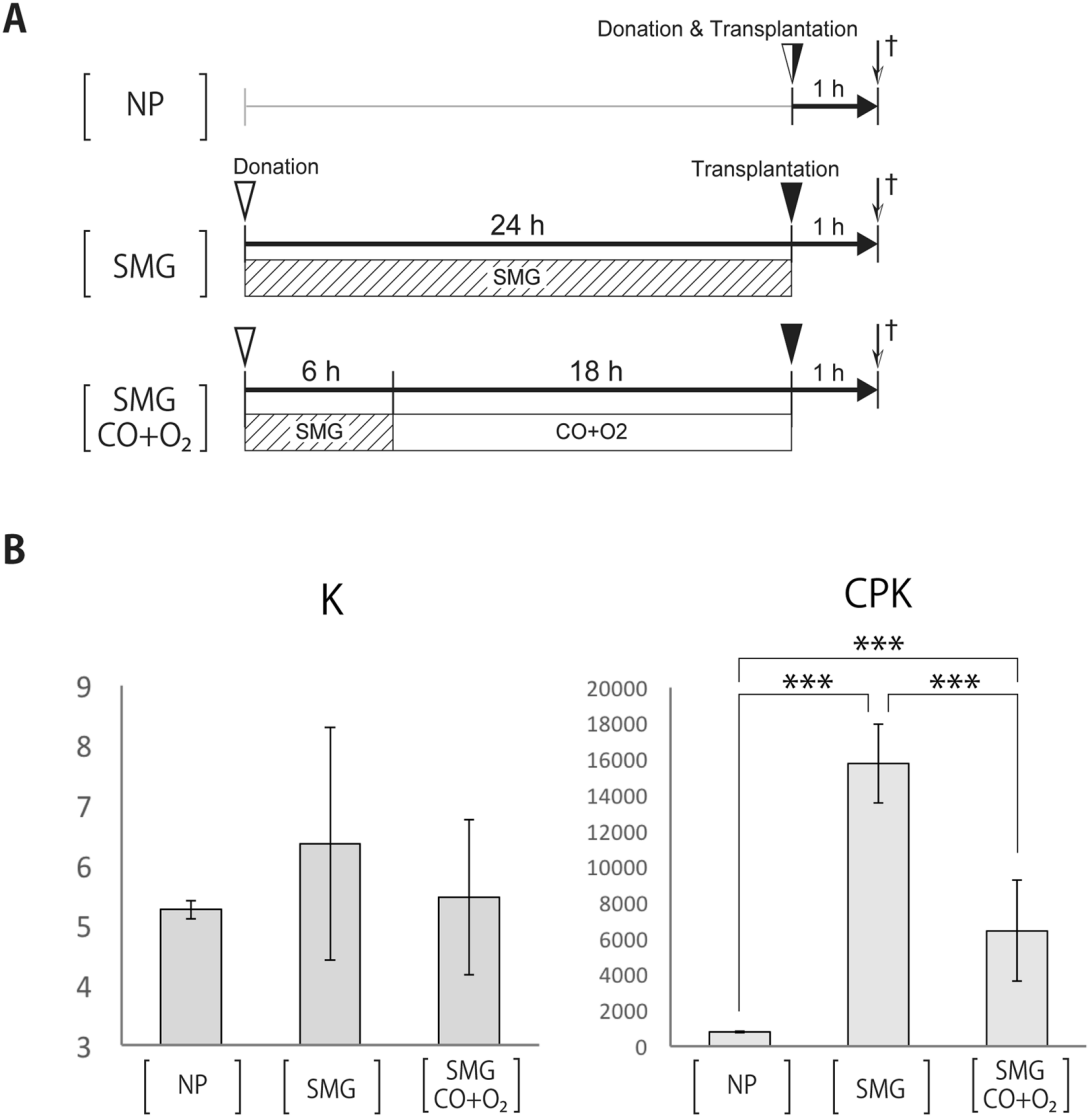
Evaluation of transplanted limb damage per group. **(A)** Experimental design. **(B)** The potassium (K) and creatine phosphokinase (CPK) levels in blood samples taken from recipients after transplantation. We compared groups receiving limbs that were not preserved (NP), preserved by wrapping in saline-moistened gauze (SMG) at 4 °C group for 24 h, and preserved by wrapping in SMG for 6 h before being preserved in a hyperbaric chamber with CO and O_2_ for 18 h at 4 °C throughout. ***P < 0.001 by the Tukey–Kramer test.

To assess the potential role of hyperbaric gases to preserve limbs in current clinical settings, we compared groups receiving limbs that were not preserved, that were preserved by wrapping in SMG at 4 °C group for 24 h, and that were preserved by wrapping in SMG for 6 h before being preserved in a hyperbaric chamber with CO and O_2_ for 18 h at 4 °C throughout. Blood samples were then collected from recipients 1 h after transplantation and potassium and creatine phosphokinase levels were measured (Fig. 4B). The creatine phosphokinase but not potassium levels in the group that received CO and O_2_ after initial SMG preservation were significantly lower than those only preserved with SMG.

## Discussion

We verified the effect of hyperbaric CO and O_2_ on amputated limb preservation in rats. Although muscle mass decreased, 86% of limbs preserved for 3 days and 50% of limbs preserved for 7 days survived more than 90 days after transplantation. These results indicate that using CO and O_2_ at high pressure could greatly improve the preservation time of amputated limbs.

The blood flow of amputated rat limbs preserved for 3 and 7 days with hyperbaric CO and O_2_ were maintained with a probability of 50% or more by macroscopic diagnosis and needlestick bleeding at POD 90. Neural evaluations of these limbs at POD 90 also indicated that the motor and sensory activities did not change in comparison with the transplanted limbs immediately after amputation. Because LEW/SsN Slc rats are an inbred strain, immune rejection was not taken into consideration in this experiment. However, muscle mass significantly decreased, and both severe muscle atrophy and fibrosis were observed in the limbs preserved for 7 days, with the rats ultimately unable to walk normally. Thus, the amputated limbs preserved for 3 and 7 days with hyperbaric CO and O_2_ could be engrafted but with deteriorated function.

Recently, using histological and ^18^F-fluorodeoxyglucose positron emission tomography analyses, we revealed that rat heart function was well preserved until 24 h, but not 48 h, after storage in hyperbaric CO and O_2_ conditions^20^. Because heart function needs to be available immediately after transplantation, and because it is difficult to recover heart function, we recommended that hearts should not be preserved in hyperbaric CO and O_2_ for more than 24 h. By contrast, human extremities can be rehabilitated after replantation, and in the case of digits, few muscles are involved. This led us to consider that preservation of amputated extremities might be feasible for 3 or 7 days with our method. And treatment is orderly performed for maintaining life and the priority of operation for the amputated extremities is postponed in the cases of multiple-trauma. Therefore, the IRI is unavoidable in the replantation and it should be important for the human amputated extremities to extend the preservable time by our method in hyperbaric CO and O_2_.

Although the preservation of human amputated extremities could be extended by our method of storage in hyperbaric CO and O_2_, this method would not be readily available at or near the site of an accident. Indeed, this preservation method is complicated and requires specialist, bulky equipment. A more practical clinical scenario might involve an initial period of cold ischemia with SMG during transport to a dedicated clinical facility at which the hyperbaric gas system could be available. In this study, we showed significantly lower creatine phosphokinase levels in the blood samples of recipients who received limbs wrapped in SMG for 6 h before preservation in the chamber with CO and O_2_ gases for 18 h compared with limbs wrapped in SMG for 24 h. These results suggested the possibility for clinical applicability of the hyperbaric CO and O_2_ preservation method.

In this study, we showed that hyperbaric CO and O_2_ treatment can extend the time limit for preservation and reduce IRI. It has been reported that CO could influence signaling pathways via soluble guanylate cyclase and/or mitogen-activated protein kinase^13,14,21^ and that the inhalation of CO has antiproliferative, antioxidant, anti-inflammatory, and antiapoptotic effects^13,14^. Previous reports also indicate that in transplantation of heart, lung, kidney, liver, and intestinal allografts or xenografts, tissues subject to CO had either reduced or prevented inflammation and apoptosis^22–27^. Hyperbaric O_2_ treatment also reduces cell death by decreasing inflammatory and apoptosis signaling and by altering enzymatic antioxidant activity^17,28,29^. These results underpin the biological plausibility of the effects hyperbaric CO and O_2_ to reduce IRI in extracted organs. However, we only performed limb preservation under the same conditions we had previously used to preserve hearts^19,20^, yet it is known that the partial pressures of each gas and total pressure influence preservation. We therefore need to consider whether there is a more optimal ratio of the two gases and whether this varies by organ or limb.

Various approaches are currently available for the preservation of organs. Simple static cold storage is used primarily for heart and liver transplantation; hypothermic machine perfusion, for kidney transplantation; and the two layers method, for pancreatic transplantation^30^. Each preservation method has its own advantages and disadvantages making them appropriate to a given organ or situation. Traditionally, amputated limbs have been preserved by wrapping in SMG and placing on ice. There have been only a few reports on the development of long-term preservation methods for amputated limbs^31,32^. Preservation in hyperbaric CO and O_2_ is a different approach, and we have provided the first evidence of successful replantation of amputated rat limbs preserved for up to 7 days. Therefore, we conclude that hyperbaric CO and O_2_ therapy could be developed to preserve amputated limbs in the long term. Moreover, there have been many reports on the use of other medical gases for organ preservation, including the cell-protective effects of gases such as nitric oxide, hydrogen, hydrogen sulfide, xenon, and ozone^33^. Using these gases in hyperbaric chambers, either alone or in combination, might allow further expansion for “organ preservation”. In conclusion, the preservation of amputated extremities in hyperbaric CO and O_2_ could extend the time limits for replantation.

## Methods

### Animals

This experiment used an inbred line of LEW/SsN Slc rats (male, 10 weeks old, average weight: 230 g, range: 220–245 g, intact: *n* = 4, donors: *n* = 74, recipients: *n* = 56) that were purchased from the Shizuoka Laboratory Animal Center (Shizuoka, Japan). All handling and care of the rats conformed to the National Institutes of Health guidelines for animal research, and all experimental protocols involving animals were approved by the National Research Institute for Child Health and Development Animal Care and Use Committee (Permit Number: S-24059). All experiments involving animals were performed in accordance with the relevant guidelines and experimental protocols. All efforts were made to minimize animal suffering.

### Experimental design

**Figure 5:**
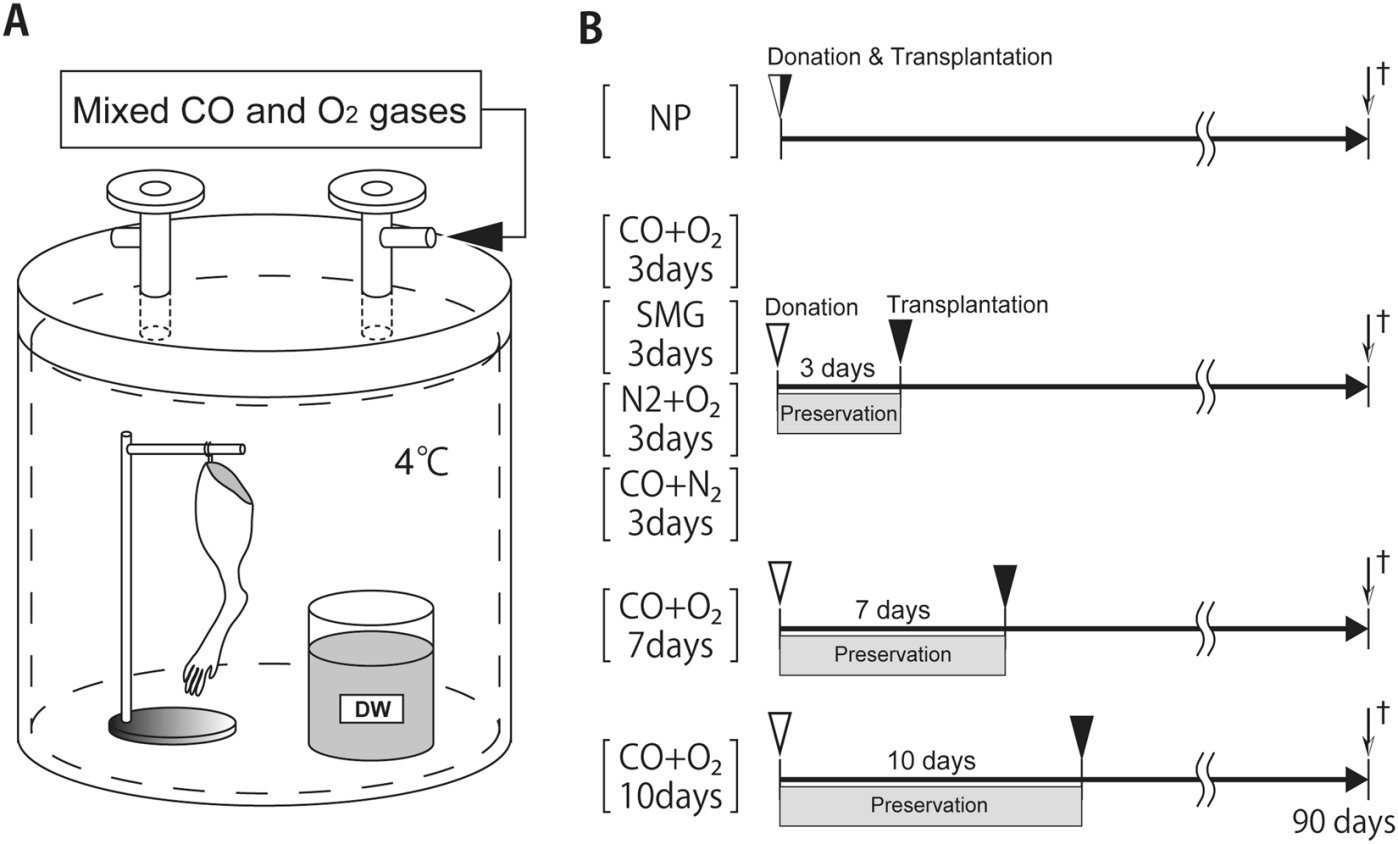
Method of limb preservation in a hyperbaric chamber. **(A)** A schematic of the preservation method. The chamber was filled with CO at 4000 hPa and PO_2_ at 3000 hPa. During limb preservation, a flask with 50 mL of distilled water (DW) was placed in the chamber to maintain humidity. **(B)** Experimental design.

Amputated rat limbs were evaluated with orthotropic hind limb transplantation. Hind limbs were amputated from the donor rats, and blood was removed from femoral artery and replaced with Krebs–Henseleit solution at 4 °C. The limb was preserved at 4 °C in the custom-built seven atmospheric pressure-resistant chamber (L: 165 mm/W: 165 mm/H: 200 mm, material: stainless, Nakamura Iron Works Co., Ltd., Tokyo, Japan) for each condition, as follows (Fig. 5):

i. Limbs were hung inside a chamber filled with a mixture of CO and O_2_ gases (7000 hPa; PCO = 4000 hPa, PO_2_ = 3000 hPa) for 3 days (CO + O_2_ 3 days group; *n* = 14), 7 days (CO + O_2_ 7 days group; *n* = 10), or 10 days (CO + O_2_ 10 days group; *n* = 6).
ii. Limbs were hung inside a chamber filled with a mixture of CO and N_2_ gases (7000 hPa; PCO = 4000 hPa, PN_2_ = 3000 hPa) for 3 days (CO + N_2_ 3 days group; *n* = 6).
iii. Limbs were hung inside a chamber filled with a mixture of O_2_ and N_2_ gases (7000 hPa; PO_2_ = 4000 hPa, PN_2_ = 3000 hPa) for 3 days (O_2_ + N_2_ 3 days group: *n* = 6).

In the preservation chamber, a flask was placed with 50 mL of distilled water to maintain humidity. Rat limbs used immediately after extraction (i.e., NP group) served as the positive controls (*n* = 8), whereas limbs wrapped in SMG for 3 days at 4 °C (i.e., SMG 3 days group) served as negative controls (*n* = 6). Each limb underwent orthotopic transplant to syngeneic recipient rats and was evaluated by comparison with intact rat limbs (Intact: *n* = 4). Alteration in transplanted limb color to black indicated graft failure, and rats were then sacrificed by cardiac incision under deep anesthesia. At the end of the experimental time, triceps surae muscles of a sample were excised. At POD 90, transplanted limbs were evaluated for the following: macroscopic diagnosis, survival rate, computed tomography, wet weights, histology, triceps surae muscle motor and sensory nerve conduction tests, and walking track analysis.

We performed further study to assess the role of hyperbaric preservation in clinical medicine (Fig. 4A). For this, we compared groups receiving limbs that were not preserved (NP group: *n* = 6), that were preserved by wrapping in SMG for 24 h at 4 °C (SMG group: *n* = 6), and that were preserved by wrapping in SMG for 6 h before being preserved in a hyperbaric chamber with CO and O_2_ (7000 hPa; PCO = 4000 hPa, PO_2_ = 3000 hPa) for 18 h at 4 °C (SMG CO + O_2_ group: *n* = 6) throughout. Blood samples were then collected from recipients 1 h after transplantation, and potassium and creatine phosphokinase levels were measured.

### Surgical procedure

Preparation of donor and recipient limbs was performed while rats were deeply anesthetized with isoflurane (ISOFLU^®^, Dainippon Sumitomo Pharma Co., Ltd.), using an inhalation anesthesia apparatus (Univentor 400 Anesthesia Unit, Univentor Ltd.). Anesthetic induction was performed in cages with a 4–5% concentration of isoflurane at a high flow rate (450–500 mL/min) for about 5 min. We then delivered an isoflurane concentration of 1.5–2.0% at 150–200 mL/min through a mask covering the rats’ noses. Donor rats were administered heparin (50 U; Ajinomoto, Tokyo, Japan) by tail vein injection as pretreatment. The skin, muscles, vessels, nerves, and bone were cut around the circumference of the left hind limb near the level of the inguinal ligament. Blood was then removed from the femoral artery and replaced with Krebs–Henseleit solution at 4 °C. In recipient rats, femur intramedullary fracture fixation was stabilized by an 18-gage needle, and the muscles were sutured with 4-0 Vicryl. The femoral artery and vein were anastomosed with 10-0 nylon, and the sciatic nerve was sutured with 10-0 nylon epineural sutures under a surgical microscope. Blood flow was checked after 15 min, and the skin was closed. An injection of the analgesic Rimadyl (carprofen 5%; Pfizer, UK) was administered subcutaneously (0.1 mL/kg) until POD 7, and the condition of transplanted limbs was observed daily.

### Computed tomography

Computed tomography analyses were performed with 0.65 mm slices using an Optima CT660 (GE Healthcare, Milwaukee, WI, USA) equipped with an X-ray tube working at 120 kV/100 mA.

### Motor and sensory nerve conduction tests

All electrophysiological measurements were taken from the left hind limbs. Electrophysiological measurements were recorded using an electromyography/evoked potential measuring system (Neuropack S1 MEB-9400, Nihon Kohden Corp., Tokyo, Japan). Supramaximal stimuli were applied for all studies, and the velocities were calculated from the onset latency of the recorded potential. All sensory studies were digitally averaged until a clear waveform was obtained. Motor nerve conduction tests were performed by placing cathode needle electrodes in two positions (the sciatic notch and approximately 3 cm distally). The recording needle electrode was placed in the foot. Orthodromic sensory studies were performed using the motor recording electrode, described as the stimulating electrode above, with recording performed at the sciatic notch.

### Walking track analysis

Walking track analysis was performed to examine replanted hind limb at POD 90. Briefly, rats were allowed conditioning trials in a walking track (8.2 × 42 cm) darkened at one end. White office paper cut to the appropriate dimensions was placed on the bottom of the track. The rat’s hind limbs were dipped in Chinese ink, and the rat was permitted to walk down the track, leaving its hind footprints on the paper. In addition, walking appearance was recorded with a digital camera (COOLPIX S8200; Nikon Corporation, Tokyo, Japan).

### Light microscopy

Hind limbs from each group were fixed in Bouin’s solution for 3 h. The samples were then washed, dehydrated with an ethanol series, and embedded in paraffin. Serial 6 μm sections were cut with a microtome and stained with Gill’s hematoxylin III and 2% eosin Y.

### Serum biochemistry

For the serum biochemical evaluation the following levels were determined: potassium (K) and creatine phosphokinase (CPK).

### Statistical analysis

Data are presented as means ± standard deviations, and significance differences were determined by analysis of variance with post-hoc tests. A *p*-value < 0.05 was considered statistically significant.

### Data availability

All data generated during this study are included in this published article.
